# Malignant Tourette’s Syndrome in an Adult on Deep Brain Stimulation Presenting With Rhabdomyolysis

**DOI:** 10.7759/cureus.44436

**Published:** 2023-08-31

**Authors:** Varun Monga, Rohit Madan, Niraj Arora

**Affiliations:** 1 Psychiatry, Banner Health, Phoenix, USA; 2 Psychiatry, University of Arizona College of Medicine, Tucson, USA; 3 Neurology, University of Missouri, Columbia, USA

**Keywords:** rhabdomyolysis, deep brain stimulation, adult, tics, malignant tourette

## Abstract

Tourette's syndrome (TS) patients experiencing severe tics and behavioral disturbances can have a rare complication called rhabdomyolysis (RML), which is characterized by the breakdown of muscle tissue. The occurrence of RML poses a significant physical and emotional risk to patients with TS by impacting the quality of life and in some cases causing severe damage. In this case report, we present the first documented case of RML resulting from severe tics in an adult with a diagnosis of TS. The patient exhibited severe tics and self-injurious behaviors that led to elevated creatine kinase and a subsequent diagnosis of RML requiring hospitalization with a complex hospital course. The patient did not have neuroleptic malignant syndrome as his laboratory parameters improved with the decrease in severity of tics. Our case highlights the potential complication of RML because of severe tics independent of neuroleptic drug use in a patient with TS.

## Introduction

Tourette's syndrome (TS) as defined by DSM-5 is a neurodevelopmental disorder characterized by the presence of both motor and phonic tics that appear before the age of 18 and persists for a duration of 12 months with no other underlying condition [1]. According to a systematic review that included 21 population-based studies focusing on children between the ages of 4 and 18, the estimated prevalence of TS was found to be 0.52%. It is more common in males with a male-to-female ratio of 3-4:1 [2]. It is an uncommon disorder in adulthood with prevalence falling to approximately 0.001% (118 cases in a million) [3]. The prevalence can vary among studies due to differences in the study population, methodology, and sample size. TS can have a significant impact on the quality of life not only due to the debilitating tics but also due to its secondary association with obsessive-compulsive disorder (OCD), obsessive-compulsive behaviors (OCB), and attention-deficit hyperactive disorder (ADHD) [4]. Treatment options for TS encompass a range of approaches including behavioral, pharmacological, or surgical interventions. Behavioral interventions commonly used for TS include exposure and response prevention, habit reversal techniques, and comprehensive behavioral intervention. Pharmacological interventions for TS may involve the use of medications such as alpha-2 adrenergic agonists such as clonidine, antiepileptic drugs such as topiramate, and dopamine receptor blocking agents like tiapride and ecopipam. Antipsychotics also have efficacy in this condition; haloperidol, pimozide, and aripiprazole are currently the only medications approved by the U.S. Food and Drug Administration (FDA) to treat tics. Vesicular monoamine transporter type 2 inhibitors (VMAT2) such as tetrabenazine are also being used. Cannabinoids and botulinum toxin have demonstrated promising potential however their efficacy and safety have not been extensively investigated. Deep brain stimulation (DBS) seems to be the surgical intervention of choice for TS [5]. There are several case reports demonstrating the efficacy of electroconvulsive therapy for TS, but there are no controlled studies, and most are complicated by severe psychiatric illness [6]. The Yale Global Tic Severity Scale (YGTSS) is a clinician-administered scale used to measure the severity and impact of tics in patients with TS and other tic disorders [7].

While it is possible to develop RML with TS, it is extremely uncommon. To date, there have been only two cases reported of TS with rhabdomyolysis in adolescent patients however there is no case report of RML with malignant TS in adults [8]. In our case, an adult with TS on DBS for years presented with worsened severe motor tics and behavior disruptions that caused RML. RML from TS can be challenging to manage in a clinical setting. Since there are no specific guidelines for the management of such cases due to overall small incidence, the case can serve as a learning experience for physicians and intensivists where benign symptoms can lead to several complications causing prolonged hospital stays. 

## Case presentation

**Figure 1:**
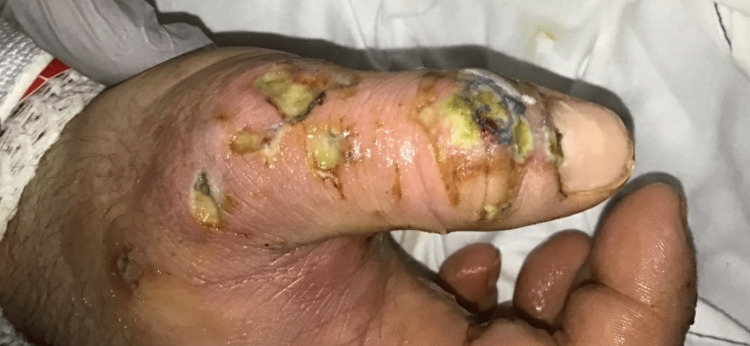
Wounds on hands

A 20-year-old male with a history of TS, ADHD, OCD, OCB, and anxiety with depression presented with progressive worsening of verbal/motor tics and diaphoresis for the last several months. Tics started when the patient was six years old with echolalia and coprolalia. There is no history of developmental disorders or cognition issues. There is no family history of substance abuse, tic disorder, ADHD, or depression. DBS placement was done four years ago for the management of tics with partial improvement. The mother noted an increase in the motor tics in the form of fists, legs, and body slamming along with mouth clenching. Vocal tics manifested as severe grunting sounds with loud forceful noises. Biting in the form of chewing on something (cloth piece, hard plastic) was noted. The YGTSS score was determined to be 99 based on the severity of tics and overall presentation. Admission to the integrated medicine service with psychiatry follow-up was considered for management. He was afebrile on admission with a temperature of 36.9°C, a heart rate of 79/min, a blood pressure of 132/76mmHg, and SpO_2_ of 99% on room air. Neurological exam was consistent with anxious mood and affect, but the patient was cooperative for examination, and bilateral hands, foot, and scalp had abrasions with dried blood (Figure 1). The outpatient regimen included sertraline 100mg daily, olanzapine 10mg at bedtime, clonidine 0.2mg twice daily, clonazepam 2mg twice daily, and hydroxyzine 50mg as needed for anxiety. 

Investigations

**Figure 2:**
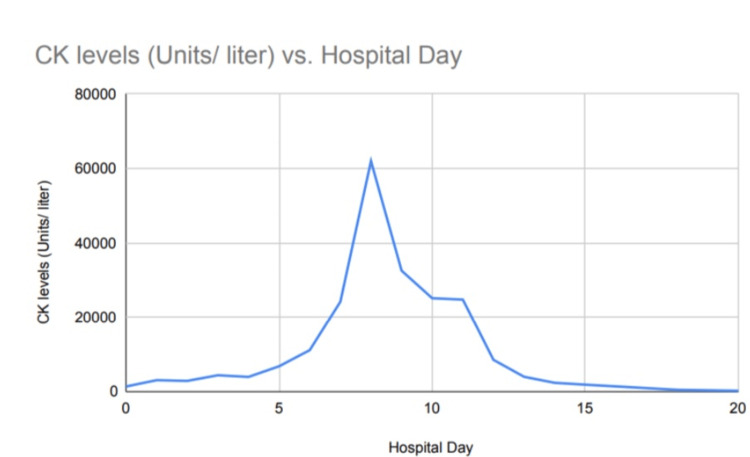
Trends of CK during the hospital stay in the ICU CK: Creatine kinase; ICU: intensive care unit

During his hospitalization, the creatine kinase (CK) level increased to 61,992 units/ liter (Figure 2). Serum creatinine was mildly elevated (1.3mg/dl) on admission, Liver function tests were normal during admission but worsened during the intensive care unit (ICU) stay but the rise was not significant (x3 upper limit of normal). Serum ceruloplasmin levels were normal. Urine drug screen, thyroid function tests, autoimmune, and paraneoplastic panel were negative. Lactic acid and pyruvic acid levels were within normal limits. Urine tests for porphyria were negative. Poor function of cytochrome P450 CYP2D6 drug-metabolizing enzyme was known based on prior testing.

Hospital course and follow-up

During the first seven days of hospital admission, anxiety, agitation, and violent behavior episodes from motor tics continued despite management with oral sertraline 100mg daily, clonidine 0.2mg twice daily, clonazepam 2mg three times a day, melatonin 15 mg at bedtime, valproic acid 500mg twice daily, and risperidone 1mg at bedtime. CK levels continue to trend despite the use of normal saline (4 liters/day) intravenously while the patient continues to have violent self-inflicting behavior causing multiple wounds all over his body and lack of sleep throughout due to motor movements. At this time, decision to transition care under the ICU was considered for the need for dexmedetomidine infusion. After the dexmedetomidine infusion up to a maximum rate of 1.2mcg/kg/hour, there was improvement in sleep for the next two days with a decrease in violent motor movements. CK levels decreased gradually over the next few days with almost a return to baseline after about a week (Figure 2). Quetiapine 25mg three times a day was added along with trazodone 50mg twice daily for sleep while the dexmedetomidine infusion was weaned off. There was a return of motor tics without aggressive violent behavior. The battery for DBS (centromedian nucleus of the thalamus) was replaced without any change in the motor tics despite adjustment of the settings in impedance. Elective intubation along with sedation with midazolam and fentanyl infusion and subsequently pentobarbital infusion was considered for management of tics to help with the wound healing for 7-10 days. During this period, oral tetrabenazine 12.5mg twice daily, up titrated to 25 mg three times a day with weekly up titration, was initiated to control the tics. After weaning from pentobarbital sedation and extubation, there was severe apathy which was attributed to the effect of tetrabenazine. After four days of extubation, there was worsening of respiratory status (hypoxemia, tachypnea with inability to protect the airways) which required re-intubation. The patient developed severe sepsis and broad-spectrum antibiotics were considered. This prolonged his hospitalization stay which was further complicated by ventilator-associated pneumonia, lung abscess due to Klebsiella pneumoniae, necrotizing pneumonia, bacteremia with staphylococcus and enterococcus species, stress-related duodenal ulcer with upper gastrointestinal bleed, and severe cachexia with poor nutritional status. Tracheostomy for mechanical ventilation management and a gastrostomy tube for feeding were placed. During this time, which took several weeks judicious sedation with combination of propofol, fentanyl, and midazolam was used intermittently with careful monitoring of CK levels. Skin wounds all over the body were healed however, motor tics continued once the sedation was weaned off and the patient was discharged to an inpatient rehabilitation facility. Discharge medications included quetiapine 50 mg at bedtime, clonidine 0.1 mg three times a day, sertraline 200mg daily, gabapentin 200 mg three times a day, and clonazepam 0.5mg twice a day. On a two-month follow-up visit, the patient was discharged to home and able to ambulate and gain body weight appropriately, continuing to have motor tics with anxiety and depression symptoms.

## Discussion

TS emerges during childhood and is often accompanied by various psychiatric conditions such as ADHD, OCD, anxiety, and mood disorders. The combination of these symptoms often leads to substantial impairment in daily functioning and in some cases can lead to disability [9]. In certain instances, these tics may persist into adulthood and prove to be resistant to treatment, leading to significant complications. This is often referred to as malignant TS. 

One incidence study from a movement disorder clinic in the United States defined malignant TS as at least two emergency room (ER) visits or at least one hospitalization due to TS or its associated behavioral complications. 5.1% of the individuals met the criteria for malignant TS. The reasons for hospitalizations and ER visits included tic-related behavior, self-injurious behaviors (SIB), violence, and suicidal ideations or attempts. When compared to patients with nonmalignant TS, those diagnosed with malignant TS were more prone to have psychiatric comorbidities such as OCB/OCD, complex phonic tics, coprolalia, copropraxia, SIB, mood disorder, and life-threatening symptoms. Furthermore, it tends to be more resistant to medical treatment [10].

Some complications of malignant TS described in the literature include cervical myelopathy, cervical disc herniation with spinal cord compression, vertebral artery dissection with stroke, and bilateral self-induced retinal detachment [11,12]. There are two reported cases of rhabdomyolysis in adolescents secondary to tic fits [8]. Severe tics in association with psychiatric disorders in the form of mood dysregulation and behavioral disturbances in the form of self-injurious behaviors can exacerbate injury to the muscles that can cause rhabdomyolysis as noted in our case. 

RML is a clinical syndrome characterized by acute muscle weakness, myalgia, and increased CK levels of more than 1000 IU/L or > 5 times the upper limit of normal. Myoglobinuria and acute kidney injury may (AKI) be present. It is typically caused by physical injury, extreme muscle exertion, certain medications, and drug use. The most critical complication is an AKI, which can lead to cardiac arrhythmia due to hyperkalemia, and is frequently accompanied by coagulopathy [13].

Identification of the specific causes and the use of appropriate countermeasures directed at the triggering events, including discontinuation of drugs or other toxins that may be etiologic factors. In our case, no nephrotoxic drugs were initiated which could have been attributed to AKI and RML. We ruled out neuroleptic malignant syndrome (NMS), serotonin syndrome, hypothyroidism, malignant hyperthermia, paraneoplastic syndrome, and DBS malfunction. Laboratory work-up ruled any illicit drug intoxication or withdrawal, central nervous system or systemic infections, trauma, pheochromocytoma, or porphyria.

Our patient was on olanzapine 10 mg which did not meet the criteria for NMS, lacking rigidity, hyperthermia, and autonomic dysfunction, and was taking SSRI sertraline 100 mg daily but did not have tremors, hyperreflexia, and clonus seen in serotonin syndrome. DBS was assessed for optimal functioning and the battery was replaced as abundance of caution with settings adjusted with no relief. We determined that treatment of refractory tics with behavioral disturbances presenting as self-injurious behavior was the cause of RML in our case. 

In our case, we started with supportive treatment with IV fluids, changing antipsychotic to low-dose risperidone 1 mg and adding valproic acid 500 mg twice a day but symptoms (motor tics, self-injurious behaviors) and RML continued. Switched to dexmedetomidine infusion which led to CK trending down and was switched to quetiapine 25 mg twice a day from risperidone 1 mg and trazodone 50 mg twice a day. As we weaned off dexmedetomidine infusion, there was a return of motor tics without aggressive self-injurious behaviors. This was a concern due to extensive wounds that motor tics were interfering with their healing. At this point, DBS was reassessed for optimal functioning and the battery was replaced with no change in severity of motor tics. Trial of VMAT2 inhibitor, tetrabenazine 25 mg BID dose led to serious apathy given cytochrome P450 CYP2D6 poor metabolizer status of our patient and had to be discontinued. 

Given no change in motor tics, pentobarbital infusion was started which led to very serious complications secondary to hypoxia. As we managed complications over several weeks of hospitalization, this allowed for wounds to heal. The patient was eventually discharged to an inpatient rehabilitation facility as wounds healed, and self-injurious behaviors were remitted although motor tics continued. 

To our knowledge, there are no prior reports of usage of pentobarbital coma for the treatment of tics and we would presently not recommend its routine use given several complications our patient had. Also, the use of valproate for tics showed promise in several reported case series, but this benefit did not carry through when a systematic review and meta-analysis was conducted [14] and thus, it is not recommended for routine use in TS. We discontinued it on our patient prior to discharge. 

Management of refractory tic disorder complicated with behavioral disturbances can be challenging especially when systemic insult in the form of RML is co-existing. Quick resolution of triggering insults for RML is crucial to prevent serious complications and mortality. There is very limited information as to how to manage TS with RML. 

## Conclusions

Malignant TS in an adult with behavioral disturbances in the form of SIB can cause RML independent of neuroleptic use. Clinicians need to be aware of this rather rare but serious complication of severe tics. Low levels of cytochrome P450 CYP2D6 drug-metabolizing enzyme can be a barrier to using VMAT-2 inhibitors as they can cause severe dopamine depletion and cause apathy. The key is identifying triggers for behavioral disturbances and managing them simultaneously as the clinician attempts to control tics.
